# Changes in composition, ecology and structure of high-mountain vegetation: a re-visitation study over 42 years

**DOI:** 10.1093/aobpla/plw004

**Published:** 2016-01-27

**Authors:** Alberto Evangelista, Ludovico Frate, Maria Laura Carranza, Fabio Attorre, Giovanni Pelino, Angela Stanisci

**Affiliations:** 1 Envix-Lab, Dipartimento di Bioscienze e Territorio, Università degli Studi del Molise, Contrada Fonte Lappone, 86090 Pesche (IS), Italy; 2 Istituto di Biologia Agro-Ambientale e Forestale, CNR/IBAF, Monterotondo, 00015 Rome, Italy; 3 Dipartimento di Biologia Ambientale, Università di Roma “La Sapienza”, Piazzale Aldo Moro 5, 00185 Rome, Italy

**Keywords:** Global change, Landolt indicators, life forms, Mediterranean mountains, phytosociological relevés, re-visitation study

## Abstract

High-mountain ecosystems are increasingly threatened by climate change, causing biodiversity loss, habitat degradation and landscape modifications. However, very few detailed studies have focussed on plant biodiversity in the high mountains of the Mediterranean. In this study, we investigated the long-term changes that have occurred in the composition, structure and ecology of high-mountain vegetation in the central Apennines (Majella) over the last 42 years. We performed a re-visitation study, using historical and newly collected vegetation data to explore which ecological and structural features have been the most successful in coping with climatic changes. Vegetation changes were analysed by comparing geo-referenced phytosociological relevés collected in high-mountain habitats (dolines, gentle slopes and ridges) on the Majella massif in 1972 and in 2014. Composition analysis was performed by detrended correspondence analysis, followed by an analysis of similarities for statistical significance assessment and by similarity percentage procedure (SIMPER) for identifying which species indicate temporal changes. Changes in ecological and structural indicators were analysed by a permutational multivariate analysis of variance, followed by a post hoc comparison. Over the last 42 years, clear floristic changes and significant ecological and structural variations occurred. We observed a significant increase in the thermophilic and mesonitrophilic plant species and an increment in the frequencies of hemicryptophytes. This re-visitation study in the Apennines agrees with observations in other alpine ecosystems, providing new insights for a better understanding of the effects of global change on Mediterranean high-mountain biodiversity. The observed changes in floristic composition, the thermophilization process and the shift towards a more nutrient-demanding vegetation are likely attributable to the combined effect of higher temperatures and the increase in soil nutrients triggered by global change. The re-visitation approach adopted herein represents a powerful tool for studying climate-related changes in sensitive high-mountain habitats.

## Abstract (precis)

Mediterranean high-mountain ecosystems are increasingly threatened by climate change, causing biodiversity loss, habitat degradation and landscape modifications. In this work, we used phytosociological relevés to conduct a re-visitation study in order to analyze changes in floristic composition over the last 42 years in the central Apennines (Majella National Park). We observed changes in floristic composition, along with a significant increase in thermophilic and nutrient-demanding species. Such changes are likely attributable to the combined effect of higher temperatures and the increase in soil nutrients triggered by global change.

## Introduction

High-mountain ecosystems are increasingly threatened by climate change, causing biodiversity loss, habitat degradation and landscape modifications (e.g. Körner 2003; Bruun *et al*. 2006). Mountain habitats in Europe contain ∼20 % of the native flora (Väre *et al*. 2003) and are authentic hotspots of plant diversity, hosting highly specialized vascular plants (Barthlott *et al*. 1996; Myers *et al*. 2000) and many endemics (Pauli *et al*. 2007). These ecosystems are sensitive to climatic factors and respond differently according to the rate of climatic change, the species pool and the biogeographical region (Beniston 2003; Pauli *et al*. 2012). Among these mountain habitats, the Mediterranean mountains in Europe deserve particular attention. They are represented by a few isolated peaks, which constituted the major refugia of plant species during the ice age of the Pleistocene, and at present, they host a high number of endemic and rare plants (Pauli *et al*. 2003; Kazakis *et al*. 2007; Stanisci *et al*. 2011; van Gils *et al*. 2012; Gutiérrez Girón and Gavilán 2013). Moreover, the orographic discontinuity of the Mediterranean mountains makes them particularly vulnerable to biodiversity loss (Nogués Bravo *et al*. 2008).

The last Intergovernmental Panel on Climate Change (IPCC) clearly noted the anomalous rates of change occurring in many environmental parameters such as temperature, atmospheric moisture content and atmospheric nitrogen (N) deposition (IPCC 2014). For example, the period between 1983 and 2012 was the warmest 30-year period of the millennium in the northern hemisphere. In Italy, during the period 1955–2005, the mean temperatures increased by 1.6 °C in spring and of 1.9 °C in summer (Brunetti *et al*. 2006; Toreti and Desiato 2008). Besides the increase in average temperatures, the IPCC (2013) also reported an increase in the atmospheric moisture content, which was related to recent changes in precipitation patterns and with the increment in evapotranspiration rates. Yet, atmospheric N deposition increased in Europe in recent decades, reaching rates 20 times higher than before the Industrial Revolution (e.g. Vitousek *et al*. 1997; Bobbink *et al*. 2010).

Global change processes attracted the interest of many ecologists, and a rich scientific literature addresses their effects on biodiversity at different spatial and temporal scales (see Parmesan 2006; Bellard *et al*. 2012). For instance, the increase of soluble N deposition, recently recorded in high-mountain habitats (Hättenschwiler and Körner 1997; Tørseth and Semb 1997), has been reported to limit plant growth and diversity in terrestrial ecosystems (Vitousek and Howarth 1991; Gong *et al*. 2015) and may restrict plant growth in alpine species (Hiltbrunner *et al*. 2005). Other studies indicated that early melting snow promotes the increment of soil moisture values at the beginning of the vegetative period with consequences on community composition, species richness and the occurrence patterns of individual species (Körner 2003; le Roux *et al*. 2013).

Pauli *et al*. (2012) documented two different trends in European mountains regarding the relation between vascular plant species and climate warming in a short-term study (7 years). In particular, in the northern mountains, a rapid increase in species richness occurred, whereas in the Mediterranean areas, where both climatic conditions and local biodiversity are considerably different from temperate and boreal regions, species richness decreased. Moreover, Gottfried *et al*. (2012) identified a consistent thermophilization process on the high-mountain vegetation (7 years). Consistent changes in plant community composition (e.g. species richness and diversity) and ecology (e.g. the upward shifting of thermophilic plant species) have been described in many central European mountains, namely in the Alps through both short-term (∼7–10 years) (e.g. Pauli *et al*. 2007, 2012; Erschbamer *et al*. 2009, 2011) and long-term (∼50–100 years) (e.g. Körner 2003; Cannone *et al*. 2007; Holzinger *et al*. 2008; Parolo and Rossi 2008; Vittoz *et al*. 2008; Britton *et al*. 2009; Grabherr *et al*. 2010; Engler *et al*. 2011; Matteodo *et al*. 2013) vegetation analyses. Despite the peculiarities present in terms of the vegetation that characterize Mediterranean mountain habitats and their sensitivity to climatic variations, few studies have described the effects of climatic changes on Mediterranean high-mountain plant communities, and these studies mainly addressed a relatively short time period (Petriccione 2005; Fernández Calzado and Molero 2013; Stanisci *et al*. 2014). Instead, studies focussing on long-term changes in such fragile ecosystems are almost absent (Jiménez-Alfaro *et al*. 2014). Long-term studies represent a unique and valuable way for identifying large-scale vegetation patterns over time and for predicting how a changing climate affects the biodiversity of mountain habitats (Stöckli *et al*. 2011; Matteodo *et al*. 2013), and thus, these long-term studies represent an area of research that deserves attention, especially in the Mediterranean region.

In this work, we performed a re-visitation study using historical and newly collected vegetation data to explore which ecological and structural features have been most successful in coping with climatic changes during the last 42 years. The research site is part of the European Long-Term Ecological Research (LTER) network (http://www.lter-europe.net) and GLORIA project network (http://www.gloria.ac.at), where ecological research is carried out at regular intervals for bio-monitoring purposes.

We addressed the following questions. (i) Have the abundance and distribution of vascular plant species of high-mountain habitats changed during the last 42 years? (ii) Have certain plant structural or ecological characteristics been favoured under the ongoing climate change?

To address these issues, we used a set of ecological plant indicators (Landolt *et al*. 2010) and life form frequencies to compare historical and present-day re-survey data.

## Methods

### Study area

**Figure 1.**
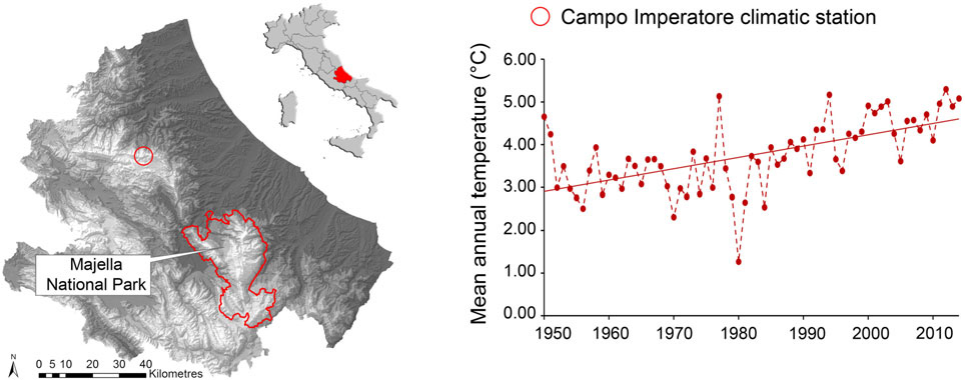
Location of the study area in central Italy. Mean annual temperatures registered in the period 1950–2014 at Campo Imperatore climatic station (2125 m a.s.l.).

The study area includes the widest high-mountain zone with alpine vegetation of the Apennines and comprises the higher sectors (from ∼2400 up to 2790 m above sea level (a.s.l.)) of the Majella National Park (Fig. 1). The area is characterized by a large limestone summit plateau modelled by a periglacial phenomenon: tectonic-karst depressions all surrounded by slopes (Giraudi 1998). The land-use history of the study area is characterized by grazing activities (mainly by sheep) that peaked in the middle of the 19th century (Palombo *et al*. 2013). Grazing pressure decreased after the second world war, due to the abandonment of transhumance practices and still decreased in the last decades because of the establishment of a protected area, the Majella National Park (van Gils *et al*. 2012). Currently, grazing intensity is very low at high elevation habitats due to the limited accessibility and low vegetation cover.

### Temperature changes: 1950–2014

To identify the climatic trends of the last 60 years, we analysed annual mean temperature data from the Campo Imperatore weather station at 2125 m a.s.l. (∼50 km away from the study area but with similar environmental conditions). During the period between 1950 and 2014, we detected a significant increase in mean annual temperature of 1.7 °C (*R*^2^ = 0.3635, *P* < 0.001), amounting to 0.26 °C per decade (Fig. 1).

### Data collection

We obtained data from 31 phytosociological relevés that had been sampled in 1972 (Feoli-Chiapella and Feoli 1977). We only selected data from relevés accompanied by an accurate description of the localities and mapped by the authors. In 2014, we re-visited the same areas and sampled 33 new phytosociological relevés **[see** **Supporting Information—Table S1****]**. Since no permanent plots were marked in the first sampling period, in the 2014 fieldwork, we re-visited the same areas by following the description of the locations, which were marked in historical vegetation maps (Feoli-Chiapella and Feoli 1977), and collected detailed information on the morphology, soil, aspect and slope reported in the reference studies (Stöckli *et al*. 2011). Relevés were conducted in three habitat types widely distributed in the analysed mountains (Stanisci *et al*. 2011): dolines, gentle slopes and ridges (see Table 1). Relevés were performed following the same sampling protocol (considering plant community type, habitat type, plot size, previous species lists and dominant species cover estimations) (Chytrý *et al*. 2014) and in the same season of the previous study in order to remove the effects of phenological differences (Vymazalová *et al*. 2012). Sampling size varied according to the habitat type (from 15 to 100 m^2^) but was the same within each community type. The plant communities were sampled using the classic phytosociological approach using the Braun-Blanquet scale of abundance/dominance (Braun-Blanquet 1964; Westhoff and van der Maarel 1973). We used Conti *et al*. (2005) as a taxonomic reference.

**Table 1.** Synthetic description of the environmental units (over 2000 m a.s.l.) of the Majella massif (central Apennines). Rel, number of relevés; Elev, elevation ± standard error (m a.s.l.); Slo, slope ± standard error (%); Veg, vegetation cover ± standard error (%); Area, reliefs area ± standard error (m^2^); Rich, species richness ± standard error (no. of species).

	Rel	Elev	Slo	Veg	Area	Rich	Characteristic plant species
Dolines	32	2493 ± 14	7 ± 1.1	87 ± 1.9	24 ± 3.9	18 ± 0.9	*Plantago atrata* and *Trifolium thalii*
Gentle slopes	19	2583 ± 36.5	13 ± 1.1	44 ± 5.9	58 ± 6.5	19 ± 1.5	*Carex kitaibeliana* subsp. *kitaibeliana* and *Festuca violacea* subsp. *italica*
Ridges	14	2659 ± 19.4	9 ± 1.3	65 ± 5.0	35 ± 7.6	22 ± 0.7	*Silene acaulis* and *Viola magellensis*

In order to investigate the changes in species composition, we analysed species abundance over time. Changes in the vegetation structure were examined using the life form categories of Raunkiaer (1934): chamaephytes (Ch), geophytes (G), hemicryptophytes (H), phanerophytes (Ph) and therophytes (Th). Plant species were classified according to flowering period in the following three groups: early (March–May), medium (June–August) and late (September–November) (Landolt *et al*. 2010). Finally, ecological features were measured using the Landolt indicator values (Landolt *et al*. 2010): temperature (*T*), moisture (*F*), soil nutrients (*N*) and dominance *in situ* (DG). These indicator values are based on the assumption that plants may serve as bioindicators (Landolt *et al*. 2010), as they reflect the species' requirements for environmental features. For each plant species, Landolt indices were expressed as a range of values from 1 to 5. Moreover, for the endemic Mediterranean flora, the Landolt indicator values were assigned by experts after checking the species requirements through literature data analysis (Feoli-Chiapella and Feoli 1977; Pignatti 1982; Conti 1998; Blasi *et al*. 2005; Pignatti *et al*. 2005; Stanisci *et al*. 2011). For comparing the ecological indicator values of the sampled flora for the different dates, we used weighted average (WA) values because they are reliable predictors of site conditions. Weighted average values were calculated using quantitative data (frequency of each plant group in each habitat type).

### Data analysis

We analysed a matrix of 116 species × 64 relevés with detrended correspondence analyses (DCAs) using the Bray–Curtis distance. Then, we performed an analysis of similarities (ANOSIM) through a one-way ANOSIM test (9999 permutations) to search for significant differences between habitat types and temporal groups. Analysis of similarities is a non-parametric test of significant difference between two or more groups, based on any distance measure (Clarke 1993). The distances are converted to ranks. Then, the similarity percentage procedure (SIMPER—Clarke 1993) was performed to determine which species contribute most consistently to differences between the temporal groups.

For each relevé, we calculated the mean Landolt bioindicator values of temperature, moisture, soil nutrients and DG weighted according to species frequency as follows:

$$\;\frac{\sum_{i=1}^{n}(r_{ij}\times x_{i})}{\sum_{i=n}^{n}r_{ij}}$$

where *r_ij_* is the frequency of the species *i* in the relevé *j*, and *x_i_* is the Landolt bioindicator value *x* for the species *i*. Moreover, for each relevé, the flowering period, life form and species richness values were calculated on presence/absence data. We performed a permutational multivariate analysis of variance (PERMANOVA, 9999 randomizations) based on Gower distances (Gower 1971), including the effect of the year (factor with two levels) and the habitat type (factor with five levels) as grouping variables. We also included the interaction between year and habitat types, allowing us to test whether the effect of year varied by habitat. The Mann–Whitney test was used post hoc to determine which variables (floristic, structural and ecological) were different between the old and new relevè. This is a non-parametric test, which means that the distributions can be of any shape. The two-tailed (Wilcoxon) Mann–Whitney *U*-test can be used to test whether the medians of two independent samples are different. All analyses were performed in the R statistical computing program (R Development Core Team 2014) using the Vegan package (Oksanen *et al*. 2013).

## Results

**Figure 2.**
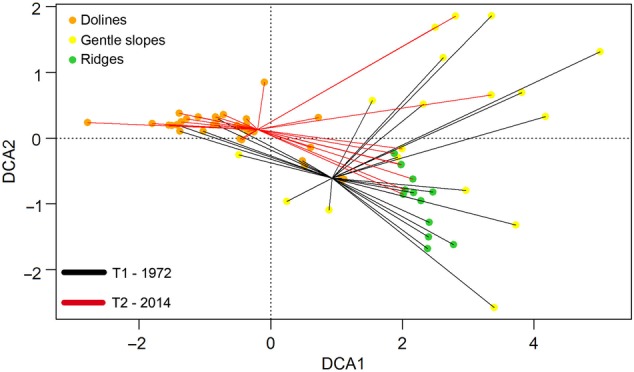
Detrended correspondence analysis scatter diagram of plots (grouped in doline, gentle slope and ridge habitat types), using species as the explanatory variables. Only the first two axes are represented. Black lines represent the relevés sampled in 1972; red lines represent the relevés sampled in 2014.

The DCA revealed differences in the floristic composition of the relevés depending on the time and the habitat types in which they were collected. Eigenvalues for the DCA axes were 0.820 for DCA1 and 0.528 for DCA2, thus clearly discriminating between the two different time periods. The first axis mainly showed the separation of the relevés in habitat types (Fig. 2), whereas the second axis reflected the temporal split between the old (upper group) and new (lower group) samples (Fig. 2). The analysis of similarity confirmed these trends and revealed significant differences among the habitat types (ANOSIM *R* value = 0.714, *n*_doline_ = 32, *n*_gentle slopes_ = 19, *n*_ridges_ = 14, *P* = 0.001) and between the temporal groups (ANOSIM *R* value = 0.302, *n*_old_ = 31, *n*_new_ = 33, *P* = 0.001). In accordance with the similarity percentage analysis (Table 2), 27 of the 116 species contributed to 50 % of the observed temporal differences in vegetation composition. Of these, 16 increased and 11 decreased over time.

**Table 2.** Plant species contribution (Species contrib. %) to the observed differences between plant communities in the two temporal groups assessed by similarity percentage procedure (SIMPER—Clarke 1993). Life forms (H, hemicryptophytes; Ch, chamaephytes; G, geophytes) and Landolt ecological indicators (*T*, temperature; *F*, moisture; *N*, soil nutrients) are also reported and mean abundance. Endemic species are indicated with asterisks.

Species	Life form	*T*	*F*	*N*	Mean abundance (%) decrease		Species contrib. (%)
					1972	2014	
Viola magellensis*	H	1.0	1.5	2.0	0.677	0.0909	2.5
Myosotis ambigens*	H	1.5	3.0	3.0	0.581	0.273	2.053
*Draba aizoides* subsp. *aizoides*	H	1.5	2.0	2.0	0.581	0.333	2.044
*Saxifraga oppositifolia* subsp. oppositifolia*	Ch	1.0	3.0	2.0	0.516	0.303	1.996
*Silene acaulis*	Ch	1.0	3.0	1.0	0.484	0.455	1.908
*Kobresia myosuroides*	H	1.5	2.0	2.0	0.484	0.212	1.75
*Salix retusa*	Ch	1.5	3.0	2.0	0.387	0.303	1.726
*Arenaria grandiflora* subsp. *grandiflora*	Ch	2.0	2.0	2.0	0.355	0.273	1.682
Galium magellense*	H	1.5	2.5	2.0	0.387	0.242	1.613
*Gentiana verna* subsp. *verna*	H	2.5	3.0	2.0	0.258	0.242	1.527
*Gentiana brachyphylla* subsp. *brachyphylla*	H	1.0	3.0	2.0	0.419	0.0303	1.518
Species	Life form	*T*	*F*	*N*	Mean abundance (%) increase		Species contrib. (%)
					1972	2014	
*Minuartia verna* subsp. *verna*	Ch	2	2	2	0.29	0.727	2.373
*Trifolium thalii*	H	1.5	3.5	3	0.161	0.606	2.329
*Plantago atrata* subsp. *atrata*	H	1.5	3.5	3	0.355	0.727	2.233
Ranunculus pollinensis*	H	2	3.5	4	0.387	0.697	2.153
*Gnaphalium hoppeanum* subsp. *magellense*	H	1.5	3.5	3	0.452	0.667	2.037
*Festuca violacea* subsp. italica*	H	1.5	3.0	3	0.419	0.606	1.999
*Carex kitaibeliana* subsp. *kitaibeliana*	H	2	2.5	3	0.516	0.758	1.962
*Armeria majellensis* subsp. majellensis*	H	2	2	2	0.484	0.545	1.952
Cerastium thomasii*	Ch	1.5	1.5	2	0.387	0.485	1.93
Taraxacum glaciale*	H	1.5	3.5	4	0	0.424	1.775
*Crepis aurea* subsp. *glabrescens*	H	2	3.0	4	0.0323	0.455	1.765
*Anthyllis vulneraria* subsp. *pulchella*	H	4	1.5	2	0.29	0.394	1.667
*Potentilla crantzii* subsp. *crantzii*	H	2	2.5	3	0.258	0.333	1.613
Achillea barrelieri*	H	1.5	3	2	0.29	0.333	1.554
*Leontodon montanus*	H	1.5	2	2	0.258	0.303	1.477
*Viola eugeniae* subsp. eugeniae*	H	2	3	2	0	0.394	1.474

**Figure 3.**
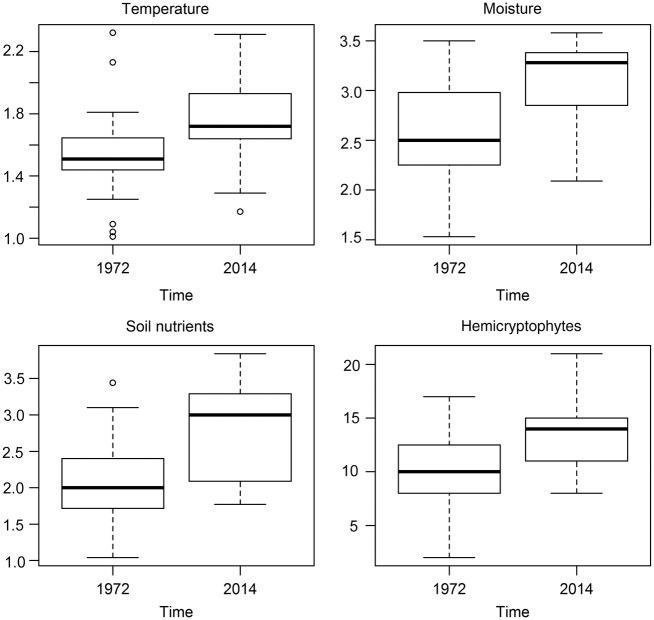
Box plots comparing the Landolt ecological values (temperature, moisture and soil nutrients) and hemicryptophyte (H) life form frequency between 1972 and 2014. All differences are significant according to a post hoc Mann–Whitney *U*-test.

The PERMANOVA test showed that the ecological and structural variables (*T*, *F*, *N*, DG, life forms, species richness and flowering period) were significantly affected by time and habitat types but not by their interaction (Table 3). In particular, post hoc Mann–Whitney *U* comparisons noted major differences between the old and new data sets for *T*, *F*, *N* and hemicryptophyte frequencies. The *T* median value increased from 1.51 to 1.72 (Mann–Whitney *z*= 3.601, *n*_old_ = 31, *n*_new_ = 33, *P* = 0.0001) (Fig. 3), variation that was due to both the decrease in frequencies of some cryophilic species (Landolt *T* values 1–1.5) and the increase in frequencies of some thermophilic species (Landolt *T* values 2–4). Ecologically, this implies an enhanced competition between slow-growing cold-adapted alpine plant species and lower elevational range species. Based on per cent similarity (Table 2), some cryophilic species, usually common in ridge habitat, have become less frequent, including *Viola magellensis*, *Myosotis ambigens* and *Silene acaulis*. Moreover, other cold-adapted species of gentle slopes, such as *Kobresia myosuroides*, *Draba aizoides* subsp*. aizoides*, *Salix retusa* and *Gentiana verna* subsp*. verna*, showed a decrease. During the same period, other species with an ecological optimum in lower vegetation belts (subalpine and treeline) and which are common in gentle slopes, such as *Minuartia verna* subsp. *verna*, *Ranunculus pollinensis*, *Armeria majellensis* subsp. *majellensis* and *Carex kitaibeliana* subsp*. kitaibeliana*, had increased in frequency (Table 2).

**Table 3.** Permutational multivariate analysis of variance result. Time, comparison between old (1972) and new (2014) relevés. Habitat type, comparison between habitat types described in Table 1.

Permutation *N*: 9999	PERMANOVA (Gower)				
Source	Sum of squares	df	Mean square	*F*	*P*
Time	0.1732	1	0.1732	6.496	0.0001
Habitat type	0.8434	2	0.4217	15.82	0.0001
Interaction	−0.4466	2	−0.2233	−8.37	0.4432
Residual	1.5466	58	0.0267		
Total	2.1166	63

The *F* median value increased from 2.50 to 3.28 (Mann–Whitney *z* = −3.393, *n*_old_ = 31, *n*_new_ = 33, *P* = 0.0003) denoting a decrease of species that require soils with medium–low moisture content (fresh soils—Landolt *F* value 2.5) and an increase of species growing on soil with moderate moisture content (moderately moist soils—Landolt *F* value 3) (Fig. 3). Actually, some mesophilous species (Landolt *F* values 3–4) that are common in doline habitats, such as *Plantago atrata* subsp. *atrata*, *Trifolium thalii*, *Viola eugeniae* subsp. *eugeniae* and *Gnaphalium hoppeanum* subsp. *magellense*, had increased in frequency in the later relevés. The same species also grow optimally in medium fertile soils (Landolt *N* values 3–4) and contribute to an increase of the *N* median value from 2.00 to 3.00 (Mann–Whitney *z* = −3.807, *n*_old_ = 31, *n*_new_ = 33, *P* = 0.0002) (Fig. 3).

Regarding life forms, only hemicryptophytes showed significant differences between the old and new relevés, increasing from 9.90 to 13.55 (median value, Mann–Whitney *z* = −4.187, *n*_old_ = 31, *n*_new_ = 33, *P* = 0.0001), the greater part of the above-mentioned expanding species belong to this life form. However, changes in DG, species richness, length of flowering period and other life forms were not significant.

## Discussion

The re-visitation study of alpine plant communities in the central Apennines showed consistent changes in floristic composition and in structural and ecological characteristics of high-mountain vegetation in doline, gentle slope and ridge habitats, along with significant increments in the Landolt indicators of temperature, moisture and soil nutrients and an increase in hemicryptophytes (herbaceous perennial plants). Although we cannot confirm with certainty that climate warming is the primary driver of these changes, the limited human impact in the area (e.g. grazing) and the increment on temperatures recorded in the central Apennines over the last 60 years (Fig. 1) lead us to suppose that the observed ecological changes are most likely related to climate change.

The long-term vegetation analysis clearly revealed an ongoing ‘thermophilization’ process (sensu Gottfried *et al*. 2012) in the central Apennines, which was related to an increase in thermophilic plant frequency and a clear decline in several cryophilic species. Similar to observations from other studies in the Alps (Grabherr *et al*. 1995, 2001; Theurillat and Guisan 2001; Walther *et al*. 2005; Pauli *et al*. 2007; Holzinger *et al*. 2008; Parolo and Rossi 2008; Vittoz *et al*. 2008; Erschbamer *et al*. 2009; Engler *et al*. 2011; Matteodo *et al*. 2013), we registered the local expansion of thermophilic species (e.g. *A. majellensis* subsp*. majellensis*, *M. verna* subsp. *verna* and *C. kitaibeliana* subsp*. kitaibeliana*). Furthermore, as described by Pauli *et al*. (2007) for the Alps, we also registered the local contraction of many cryophilic species, mainly in gentle slope habitats (e.g. *M. ambigens*, *S. acaulis*, *V. magellensis* and *Saxifraga oppositifolia* subsp*. oppositifolia*). Similar trends have been documented in other European mountains and have been related mainly to the effects of climate change (Britton *et al*. 2009; Engler *et al*. 2011; Gottfried *et al*. 2012; Fernández Calzado and Molero 2013).

We observed changes in other ecological characteristics with vegetation that, in 2014, had become more moisture and nutrient demanding. The increment of the nutrient and moisture ecological values could be related mainly to the expansion of species typical of medium fertile soils with good moisture content coming from doline habitats (e.g. *P. atrata* subsp. *atrata* and *T. thalii*). As observed in the tundra and temperate grasslands, the observed increment in nutrient ecological values could be related to the increase in the decomposition rates caused by the rise in temperatures (e.g. Gavazov 2010; Myers-Smith *et al*. 2011; Gong *et al*. 2015) and to the increase of atmospheric N deposition (Hättenschwiler and Körner 1997; Tørseth and Semb 1997). Nitrogen deposition is well known to affect species composition and richness on alpine grasslands over Europe (e.g. Bobbink *et al*. 2010). However, N deposition in Central Italy is low compared with those reported for Northern and Western Europe (Bonanomi *et al*. 2006; Ferretti *et al*. 2014). So far, due to a lack of local data, we can simply speculate that the increase in nutrient-demanding species could be related to the increase in N deposition. Similar processes towards mesic conditions were detected in montane grasslands in the western Alps, along with consistent changes in vegetation composition and structure (Theurillat and Guisan 2001). Notably, some species of medium fertile and humid soils have wide distribution ranges; that is, they are indiscriminately distributed from the montane to the alpine belt (e.g. high Landolt *T* values) (Landolt *et al*. 2010; Güsewell *et al*. 2012; Matteodo *et al*. 2013; Venn *et al*. 2014). On the other hand, cryophilic species of dry grasslands are usually poor moisture- and nutrient-demanding taxa, and high levels of soluble N deposition limit their growth (Vitousek and Howarth 1991; Hiltbrunner *et al*. 2005). The ecological changes (thermophilization and increase in the frequencies of nutrient- and moisture-demanding species) occurring in the Apennines are most likely a result of the reduction of cryophilous drought-tolerant and poor nutrient-demanding species and the expansion of lower elevational range species; both processes are probably triggered by the ongoing global change (Huber *et al*. 2007; Engler *et al*. 2011).

The long-term analysis of the Apennine's habitats revealed consistent changes in vegetation structure. Similar to that observed in long-term analysis of the Swiss Alps (Matteodo *et al*. 2013) and of the Iberian mountains (Jiménez-Alfaro *et al*. 2014), on the Italian Apennines, hemicryptophyte frequency increased over time. Jägerbrand *et al*. (2009) observed similar increments in subarctic-alpine grasslands in correspondence with nutrient addition and increasing temperatures under experimental conditions. Accordingly, recent evidence (e.g. Spasojevic and Suding 2012; Spasojevic *et al*. 2013) suggests that variations in nutrient availability, soil moisture and temperature led to changes in the functional composition of alpine plant communities with a shift towards more resource acquisitive functional traits (e.g. hemicryptophytes with well-developed leaves). Overall, our study revealed a clear long-term change in plant species abundance patterns, with an overall large increase in graminoids (e.g. *Festuca violacea* subsp. *italica* and *C. kitaibeliana* subsp. *kitaibeliana*) and other perennial herbs (e.g. *G. hoppeanum* subsp. *magellense* and *R. pollinensis*) and the consistent decrease of cryophilic cushions plants (e.g. *S. acaulis* and *S. oppositifolia* subsp. *speciosa*) and some steno-endemic species (e.g. *V. magellensis* and *M. ambigens*).

Despite the possible limitations of the applied re-visitation sampling approach to assess changes in vegetation composition structure and ecology due to the potential mismatch between old and new relevés (Chytrý *et al*. 2014), we are confident of the efficacy of our dedicated efforts to carry out the new sampling session in the same plant communities (and habitat types) as the historical one. Nevertheless, re-visitation (long-term) studies using historical records represent the only possibility for obtaining a reliable time perspective necessary to identify large-scale patterns over the last century (Stöckli *et al*. 2011; Matteodo *et al*. 2013) and for making predictions on the future assemblage of plant communities facing global changes.

## Conclusions

Our results, based on long-term data obtained by the re-visitation of historical vegetation records, reinforce previous short-term observations for reporting changes on plant composition of high-mountain habitats related with climate in Mediterranean mountains. Furthermore, we observed a shift in the ecological values of high-mountain vegetation that showed a thermophilization process and an increase in nutrient-demanding and mesic species, typical of doline and gentle slope habitats. These changes are more likely attributable to the combined effect of higher temperatures and an increase in soil nutrients triggered by global change. This study responds to the crucial need of further long-term analysis describing vegetation changes over the last century. Such a gap in knowledge impels the development of additional research in other areas, in which, like the Mediterranean high mountains, the effect of climate changes is expected to be consistent. The herein adopted re-visitation approach might represent an adequate instrument to respond to the scarcity of a long-term series of ecological data describing natural ecosystems and the results can be gathered with existing short- to medium-term permanent observation networks (e.g. LTER). Since data from historical vegetation plots are generally available in many European countries, the re-visitation studies have a potential for application to other mountains in Italy and Europe at scales ranging from local to regional. With this in mind, we hope that several case studies will be analysed in the near future to provide long-term information for increasingly larger areas.

## Sources of Funding

This work was partially supported by the NextData project (Data-LTER-Mountain) and the GLORIA-MEDIALPS project.

## Contributions by the Authors

A.S. and M.L.C. conceived and designed the experiments. A.E., G.P. and L.F. collected the data. A.E. and L.F. analysed the data. A.E., A.S., L.F., M.L.C. and F.A. wrote the manuscript.

## Conflict of Interest Statement

None declared.

## Supporting Information

The following additional information is available in the online version of this article –

**Table S1.** Phytosociological table of new relevés.

Additional Information
