# Supplementary material for: Changes in composition, ecology and structure of high-mountain vegetation: a re-visitation study over 42 years
Source: AoB Plants. 2016 Jan 27;8:plw004. doi: 10.1093/aobpla/plw004 (PMC4770936; doi:10.1093/aobpla/plw004)
Supplement: Additional Information [file supp_8_plw004_index.html]

Changes in composition, ecology and structure of high-mountain vegetation: a re-visitation study over 42 years — Changes in composition, ecology and structure of high-mountain vegetation: a re-visitation study over 42 years — Additional Information 

# Changes in composition, ecology and structure of high-mountain vegetation: a re-visitation study over 42 years

## Additional Information

Additional Information

- Additional Information - Docx file
