# Supplementary material for: Changes in composition, ecology and structure of high-mountain vegetation: a re-visitation study over 42 years
Source: AoB Plants. 2016 Jan 27;8:plw004. doi: 10.1093/aobpla/plw004 (PMC4770936; doi:10.1093/aobpla/plw004)
Supplement: Additional Information [file supp_plw004_plw004supp.docx]

| Table 1. Phytosociological relevés carried out in doline (Do), gentle slope (Gs) and ridge (Ri) habitats . For each relevé place name and geographical coordinates (system WGS 84 UTM 33) and date are listed below. | | | | | | | | | | | | | | | | | | | | | | | | | | | | | | | | | |
| --- | --- | --- | --- | --- | --- | --- | --- | --- | --- | --- | --- | --- | --- | --- | --- | --- | --- | --- | --- | --- | --- | --- | --- | --- | --- | --- | --- | --- | --- | --- | --- | --- | --- |
| Relevé number | **1** | **2** | **3** | **4** | **5** | **6** | **7** | **8** | **9** | **10** | **11** | **12** | **13** | **14** | **15** | **16** | **17** | **18** | **25** | **26** | **31** | **32** | **33** | **27** | **28** | **29** | **30** | **19** | **20** | **21** | **22** | **23** | **24** |
| Altitudine a.s.l. (m. x10) | 250 | 249 | 252 | 250 | 256 | 251 | 249 | 249 | 245 | 246 | 247 | 240 | 237 | 239 | 237 | 236 | 236 | 241 | 263 | 260 | 257 | 249 | 255 | 272 | 277 | 277 | 271 | 268 | 261 | 256 | 257 | 263 | 265 |
| Aspect | / | NE | / | / | SE | / | E | / | / | / | SSE | / | W | / | / | / | / | / | / | / | / | / | / | / | E-NE | S | S | / | SW | / | SE | S-SW | S-SW |
| Slope (°) | 3 | 2 | / | / | 4 | / | 10 | / | / | / | 10 | / | 4 | / | / | / | / | 2 | / | / | / | / | / | 20 | 15 | / | 16 | / | 15 | / | 10 | 6 | 4 |
| Rock cover (%) | 5 | 2 | 5 | 10 | 5 | 1 | / | / | / | / | 5 | / | 2 | / | / | / | / | 5 | / | / | 25 | 5 | 2 | 3 | / | / | / | 2 | / | 5 | 5 | / | / |
| Detritus cover (%) | 20 | 20 | 20 | 10 | 10 | 35 | 15 | 10 | 5 | 5 | 15 | 15 | 5 | 15 | 20 | 5 | 10 | 10 | 50 | 30 | / | / | / | 20 | 85 | 65 | 85 | 70 | 70 | 70 | 70 | 40 | 40 |
| Relevé area (m²) | 25 | 20 | 5 | 8 | 10 | 5 | 12 | 8 | 100 | 100 | 40 | 5 | 15 | 20 | 20 | 35 | 15 | 20 | 10 | 20 | 100 | 10 | 8 | 15 | 100 | 100 | 80 | 25 | 25 | 20 | 30 | 20 | 20 |
| Vegetation cover (%) | 80 | 80 | 80 | 80 | 80 | 70 | 85 | 85 | 95 | 90 | 90 | 70 | 90 | 85 | 80 | 95 | 70 | 90 | 50 | 80 | 95 | 95 | 98 | 50 | 15 | 35 | 25 | 35 | 40 | 40 | 40 | 70 | 70 |
| Habitat type | Do | Do | Do | Do | Do | Do | Do | Do | Do | Do | Do | Do | Do | Do | Do | Do | Do | Do | Do | Do | Do | Do | Do | Gs | Gs | Gs | Gs | Ri | Ri | Ri | Ri | Ri | Ri |
| Total number of species for relevée | 28 | 22 | 22 | 19 | 14 | 12 | 13 | 12 | 15 | 16 | 17 | 18 | 18 | 16 | 13 | 15 | 16 | 15 | 26 | 23 | 33 | 18 | 20 | 20 | 20 | 22 | 22 | 21 | 20 | 26 | 24 | 27 | 22 |
| **Species list** |  | | | | | | | | | | | | | | | | | | | | | | | | | | | | | | | | |
| *Poa alpina* L. subsp. *alpina* | 3 | 3 | 2 | 2 | 3 | 2 | 2 | + | 2 | 2 | 3 | 1 | 2 | 2 | 2 | 2 | 2 | 3 | 3 | 1 | 2 | 1 | 2 | + | 1 | 1 | 1 | 1 | . | . | . | + | 1 |
| *Carex kitaibeliana* Degen ex Bech. | 2 | 1 | 3 | 3 | 1 | 2 | 3 | . | + | . | 3 | 2 | . | 2 | 3 | + | + | . | + | + | 2 | 2 | 1 | 3 | . | . | . | 3 | 3 | 2 | . | 3 | 2 |
| *Plantago atrata* Hoppe subsp. *atrata* | 4 | 4 | 2 | 3 | 2 | 3 | 3 | 4 | 4 | 4 | 3 | 4 | 1 | 4 | 4 | 4 | 3 | 1 | 3 | 4 | 3 | 4 | 2 | . | . | . | . | . | . | . | 1 | . | . |
| *Minuartia verna* (L.) Hiern. subsp. *verna* | + | . | + | . | + | + | 1 | + | + | 1 | + | + | + | 1 | + | . | . | + | + | . | 1 | 1 | 1 | + | + | . | . | + | . | . | 1 | 1 | + |
| *Ranunculus pollinensis* (N. Terracc.) Chiov. | 3 | 3 | + | 3 | 2 | 3 | 2 | 2 | 2 | 2 | 4 | 2 | 2 | 3 | 4 | 3 | 1 | 4 | . | 2 | + | 2 | 2 | . | . | . | + | . | . | . | . | . | . |
| *Gnaphalium hoppeanum* Koch subsp. *magellense* (Fiori) Strid | + | + | + | 1 | 1 | + | 1 | 1 | + | 2 | 1 | 1 | + | 2 | + | + | . | + | 2 | 1 | 1 | 3 | 1 | . | . | . | . | . | . | . | . | . | . |
| *Trifolium thalii* Vill. | 1 | 2 | . | 1 | 2 | 2 | 2 | 3 | 3 | 3 | 1 | 1 | 3 | 1 | 1 | 2 | 1 | 4 | . | 3 | 1 | . | 3 | . | . | . | . | . | . | . | . | . | . |
| *Festuca violacea* Gaudin subsp.*italica* Foggi, Graz. Rossi & Signorini | + | + | 1 | . | . | . | . | . | . | . | 1 | + | 1 | 2 | + | . | . | + | + | + | + | . | + | . | . | + | . | 1 | + | + | 1 | + | + |
| *Armeria majellensis* subsp *majellensis* Boiss. | 2 | 1 | + | 1 | + | + | 3 | . | . | + | 1 | 2 | . | . | . | . | . | + | 1 | 1 | 1 | 3 | 1 | + | . | . | . | . | . | + | . | . | . |
| *Viola eugeniae* Parl. subsp. *eugeniae* | 1 | 2 | + | + | . | . | . | . | . | + | + | + | . | + | . | + | + | . | 1 | 1 | + | . | . | . | 1 | 1 | 1 | . | . | . | . | . | . |
| Crepis aurea (L.) Cass. subsp. glabrescens (Caruel) Arcang. | 1 | 1 | + | 1 | . | 1 | 1 | . | . | + | + | + | . | . | . | + | + | . | 2 | 2 | + | + | . | . | . | . | . | . | . | . | . | . | . |
| *Silene acaulis* (L.) Jacq. | + | . | + | . | . | . | . | . | . | . | . | . | . | . | . | . | . | . | + | + | 1 | . | . | 3 | 1 | 2 | 2 | 2 | 2 | 1 | 1 | 1 | 1 |
| *Taraxacum glaciale* Hand-Mazz. | . | + | . | . | . | 1 | 1 | 1 | 3 | 2 | . | . | 2 | . | 1 | 4 | 1 | 2 | + | 1 | . | + | . | . | . | . | . | . | . | . | . | . | . |
| *Anthyllis vulneraria* L. subsp. *pulchella* (Vis.) Bornm. | + | . | + | + | . | . | . | . | . | + | . | + | . | . | . | . | . | . | . | . | 3 | . | . | 2 | . | . | . | 2 | 3 | 2 | 3 | 3 | 3 |
| *Trifolium pratense* L. subsp. *semipurpureum* (Strobl) Pignatti | 1 | . | . | + | + | . | . | . | . | . | 3 | + | 2 | 2 | 1 | . | + | + | . | 1 | . | . | 1 | . | . | . | . | . | . | . | . | . | . |
| *Draba aizoides* L. subsp. *aizoides* | + | . | + | . | . | . | . | . | . | . | . | . | . | . | . | . | . | . | + | . | + | . | + | + | . | . | + | . | 1 | . | + | + | + |
| *Achillea barrelieri* Ten. | . | . | + | . | . | . | . | . | . | . | . | . | . | . | . | . | . | . | 1 | + | 1 | . | + | 1 | + | 1 | 2 | . | . | . | + | . | + |
| *Arabis surculosa* A. Terrac. | . | + | . | . | + | . | . | 2 | 1 | + | + | . | 1 | . | . | 1 | 1 | . | . | . | . | 1 | + | . | . | . | . | . | . | . | . | . | . |
| *Potentilla crantzii* (Crantz) Beck ex Fritsch subsp. *crantzii* | . | + | . | . | + | + | + | + | . | . | . | + | . | . | . | + | . | . | + | . | 1 | + | . | . | . | . | . | . | . | 1 | . | . | . |
| *Thymus praecox* Opiz subsp. *polytrichus* (Borbàs) Jalas (Th. kerneri Borbàs) | + | + | + | + | . | . | . | . | . | . | + | + | . | . | . | . | . | . | + | . | 1 | . | . | . | . | . | . | . | . | + | . | + | . |
| *Leontodon montanus* Lam. | . | . | + | . | . | . | . | . | . | . | . | . | . | . | . | . | . | . | 2 | + | 1 | + | + | + | . | 2 | 2 | . | + | . | . | . | . |
| *Salix retusa* L. | . | . | 3 | 2 | . | . | . | . | . | . | . | . | . | . | . | . | . | . | . | . | . | . | 3 | 2 | 1 | . | . | 2 | 2 | 3 | 3 | . | 1 |
| *Saxifraga oppositifolia* L. subsp. *oppositifolia* | . | . | . | . | . | . | . | . | . | . | . | . | . | . | . | . | . | . | . | . | + | . | . | + | 2 | 1 | + | . | 1 | 1 | + | + | + |
| *Acinos alpinus* (L.) Moench | 1 | 1 | . | . | + | . | . | + | . | . | . | + | . | . | . | . | . | . | + | . | 1 | + | + | . | . | . | . | . | . | . | . | . | . |
| *Botrychium lunaria* (L.) Swartz | + | . | + | . | + | . | + | . | . | + | + | . | . | . | . | . | . | . | . | . | + | + | + | . | . | . | . | . | . | . | . | . | . |
| *Myosotis ambigens* (Bég.) Grau | + | . | . | . | . | . | . | . | . | . | . | . | + | + | . | . | . | + | . | . | + | . | . | . | 1 | 1 | 1 | . | . | . | + | . | . |
| *Arenaria grandiflora* L. subsp. *grandiflora* | . | . | . | . | . | . | . | . | . | . | . | . | . | . | . | . | . | . | . | . | . | . | . | + | . | + | 1 | 1 | + | + | 1 | + | 1 |
| *Bistorta vivipara* (L.) Delarbre | . | . | + | . | . | . | . | . | . | . | . | . | . | . | . | . | . | . | . | . | + | . | . | . | 2 | . | . | 1 | 2 | 2 | 3 | + | + |
| *Herniaria glabra* L. subsp. *nebrodensis* Jan ex Nyman | 1 | + | . | + | + | . | . | 1 | + | . | . | . | + | . | . | . | . | . | . | + | . | . | . | . | . | . | . | . | . | . | . | . | . |
| *Gentiana verna* L. subsp. *verna* | + | . | + | + | . | . | . | . | . | . | . | . | . | . | . | 1 | . | + | . | + | + | . | . | . | . | . | . | . | + | . | . | . | . |
| *Androsace villosa* L. subsp. *villosa* | . | . | . | . | . | . | . | . | . | . | . | . | . | . | . | . | . | . | . | . | . | . | . | . | + | . | 1 | 1 | 1 | + | + | 1 | 1 |
| *Cerastium thomasii* Ten | . | . | . | . | . | . | + | + | + | + | . | + | . | . | . | . | . | . | + | . | + | + | . | + | 1 | 1 | + | + | . | + | 1 | . | + |
| *Galium magellense* Ten. | . | . | . | . | . | . | . | . | . | . | . | . | . | . | . | . | . | . | + | 1 | . | . | . | . | + | 1 | + | + | + | . | + | . | . |
| *Helianthemum oelandicum* (L:) Dum subsp. *alpestre* (Jacq.) Ces. | . | . | . | . | . | . | . | . | . | . | . | . | . | . | . | . | . | . | . | . | 1 | . | . | + | . | . | . | 3 | 2 | 2 | 2 | 3 | 3 |
| *Edrajanthus graminifolius* (L.) A. DC.subsp. *graminifolius* | . | . | . | . | . | . | . | . | . | . | . | . | . | . | . | . | . | . | . | . | . | . | . | 1 | . | . | . | 1 | 1 | 1 | 2 | 1 | 1 |
| *Kobresia myosuroides* (Vill.) Fiori | . | . | . | . | . | . | . | . | . | . | . | . | . | . | . | . | . | . | . | . | . | . | . | 3 | . | . | . | 2 | 3 | 3 | 3 | 2 | 3 |
| *Sedum atratum* L. | . | . | . | . | . | . | . | . | . | . | . | . | . | . | . | . | . | . | . | . | + | + | + | + | . | + | 1 | . | . | + | . | . | . |
| *Oxytropis neglecta* Ten. | . | . | . | . | . | . | . | . | . | . | . | . | . | . | . | . | . | . | . | . | . | . | . | 1 | . | . | . | 1 | . | 2 | 2 | 2 | 1 |
| Taraxacum apenninum *(Ten.) Ten.* | . | . | . | . | . | . | . | . | . | . | . | . | 1 | . | 1 | . | . | + | . | . | . | . | + | . | + | + | . | . | . | . | . | . | . |
| *Thlaspi stylosum* (Ten.) Mutel | . | . | + | + | . | + | . | . | . | . | . | . | . | . | . | . | . | . | 1 | + | . | + | . | . | . | . | . | . | . | . | . | . | . |
| *Minuartia trichocalycina* (Ten. & Guss.) Grande | . | . | . | . | . | . | . | . | 2 | 1 | . | . | + | 1 | + | . | . | . | . | . | . | . | . | . | . | . | . | . | . | . | . | . | . |
| *Pedicularis elegans* Ten. | . | . | . | . | . | . | . | . | . | . | . | . | . | . | . | . | . | . | . | . | 2 | . | . | . | . | . | . | + | . | + | . | 1 | 1 |
| *Poa molineri* Balbis | . | . | . | . | . | . | . | . | . | . | . | . | . | + | 1 | . | . | . | + | . | . | . | . | . | . | . | . | 1 | . | . | . | + | . |
| Saxifraga exarata subsp. ampullacea (Ten.) D. A. Webb | . | . | . | . | . | . | . | . | . | . | . | . | . | . | . | . | . | . | . | . | . | . | . | . | 2 | 2 | 1 | . | + | . | + | . | . |
| *Valeriana saliunca* All. | . | . | . | . | . | . | . | . | . | . | . | . | . | . | . | . | . | . | . | . | . | . | . | . | . | 2 | 1 | . | 1 | . | . | + | + |
| *Luzula spicata* (L.) DC. subsp. *italica* (Parl.) Arcang. | 2 | 1 | . | . | . | . | . | . | . | . | . | . | . | + | . | + | . | . | . | . | . | . | . | . | . | . | . | . | . | . | . | . | . |
| *Rumex nebroides* Campd. | + | 1 | . | . | . | . | . | . | 1 | + | . | . | . | . | . | . | . | . | . | . | . | . | . | . | . | . | . | . | . | . | . | . | . |
| *Sagina glabra* (Willd.) Fenzl | + | + | . | . | . | . | . | . | . | . | . | . | . | . | . | 1 | 1 | . | . | . | . | . | . | . | . | . | . | . | . | . | . | . | . |
| *Alyssum cuneifolium* Ten. subsp. *cuneifolium* | . | . | . | . | . | . | . | . | . | . | . | . | . | . | . | . | . | . | . | . | . | . | . | . | 1 | + | 1 | . | + | . | . | . | . |
| *Androsace vitaliana* (L.) Lapeyr. subsp. *praetutiana* (Sünd.) Kress | . | . | . | . | . | . | . | . | . | . | . | . | . | . | . | . | . | . | . | . | . | . | . | . | . | 1 | 1 | . | . | + | + | . | . |
| *Leontopodium alpinum* Cass. subsp. *nivale* (Ten.) Tutin | . | . | . | . | . | . | . | . | . | . | . | . | . | . | . | . | . | . | . | . | . | . | . | . | . | . | . | 2 | . | 1 | + | 2 | . |
| *Helianthemum numularium* subsp. *grandiflorum* (Scop.) Schinz & Thell. | + | . | 1 | 1 | . | . | . | . | . | . | . | . | . | . | . | . | . | . | . | . | . | . | . | . | . | . | . | . | . | . | . | . | . |
| *Avenula praetutiana* (Parl. ex Arcang.) Pignatti | . | . | . | . | . | . | . | . | . | . | . | . | . | . | . | . | . | . | . | . | . | . | . | . | . | . | . | 1 | . | . | + | + | . |
| *Carduus crysacanthus* Ten. subsp. *crysacanthus* | . | . | . | . | . | . | . | . | . | . | . | + | 3 | . | . | . | . | . | . | . | . | . | . | . | . | 1 | . | . | . | . | . | . | . |
| *Chenopodium bonus-henricus* L. | . | . | . | . | . | . | . | . | . | . | . | . | 1 | . | . | . | + | . | . | + | . | . | . | . | . | . | . | . | . | . | . | . | . |
| *Erigeron epiroticus* (Vierh.) Halàcsy | . | . | . | . | . | . | . | . | . | . | . | . | . | . | . | . | . | . | . | . | + | . | . | . | + | . | . | . | . | + | . | . | . |
| *Linaria alpina* (L.) Mill. | . | . | . | . | . | . | . | . | . | . | . | . | . | . | . | . | . | . | . | . | . | . | . | . | 1 | + | 2 | . | . | . | . | . | . |
| *Oxytropis campestris* (L.) DC. subsp. *campestris* | . | . | . | . | . | . | . | . | . | . | . | . | . | . | . | . | . | . | + | 1 | . | . | . | . | . | . | . | . | . | . | . | + | . |
| *Papaver alpinum* L. subsp. *ernesti-mayeri* (Markgr.) Wraber | . | . | . | . | . | . | . | . | . | . | . | . | . | . | . | . | . | . | . | . | . | . | . | . | 2 | 1 | 2 | . | . | . | . | . | . |
| *Phleum pratense* L. | . | . | . | . | . | . | . | . | . | . | . | . | . | . | . | . | 1 | 1 | . | 1 | . | . | . | . | . | . | . | . | . | . | . | . | . |
| *Pulsatilla alpina* (L.) Delarbre subsp. *alpina* | . | . | . | + | . | . | . | . | . | . | 1 | . | . | + | . | . | . | . | . | . | . | . | . | . | . | . | . | . | . | . | . | . | . |
| *Saxifraga paniculata* Mill. Subsp. *paniculata* | . | . | . | . | . | . | . | . | . | . | . | . | . | . | . | . | . | . | . | . | . | . | . | + | . | . | . | . | . | . | . | + | + |
| *Senecio squalidus* L. | . | . | . | . | . | . | . | . | . | . | . | . | + | . | . | . | . | . | + | + | . | . | . | . | . | . | . | . | . | . | . | . | . |
| *Sedum acre* L. | 1 | + | . | . | . | . | . | . | . | . | . | . | . | . | . | . | . | . | . | . | . | . | . | . | . | . | . | . | . | . | . | . | . |
| *Saxifraga adscendens* L. subsp. *adscendens* | + | . | . | . | . | . | . | . | . | . | . | . | . | + | . | . | . | . | . | . | . | . | . | . | . | . | . | . | . | . | . | . | . |
| *Saxifraga tridactylites* L. | + | + | . | . | . | . | . | . | . | . | . | . | . | . | . | . | . | . | . | . | . | . | . | . | . | . | . | . | . | . | . | . | . |
| *Artemisia umbelliformis* Lam. subsp. *eriantha* (Ten.) Vallès-Xirau & Brañas | . | . | . | . | . | . | . | . | . | . | . | . | . | . | . | . | . | . | . | . | . | . | . | . | . | . | . | . | . | + | . | + | . |
| *Campanula scheuchzeri* Vill. | . | + | . | . | . | . | . | . | . | . | . | . | . | . | . | . | . | . | . | . | 1 | . | . | . | . | . | . | . | . | . | . | . | . |
| *Cerastium arvense* subsp. suffruticosum (L.) Hegi | . | . | . | . | . | . | . | . | . | . | . | . | + | . | . | . | + | . | . | . | . | . | . | . | . | . | . | . | . | . | . | . | . |
| *Cerastium cerastioides* (L.) Britton | . | . | . | . | . | . | . | . | . | . | . | . | . | . | . | 1 | . | + | . | . | . | . | . | . | . | . | . | . | . | . | . | . | . |
| *Dryas octopetala* L. | . | . | . | . | . | . | . | . | . | . | . | . | . | . | . | . | . | . | . | . | . | . | . | . | . | . | . | . | . | 1 | . | + | . |
| *Iberis saxatilis* L. subsp. *saxatilis* | . | . | . | . | . | . | . | . | . | . | . | . | . | . | . | . | . | . | . | . | . | . | . | . | . | . | + | . | . | . | . | + | . |
| *Pilosella lactucella* (Wallr.) P.D. Sell & C. West | . | . | . | . | . | . | . | . | . | . | . | . | . | . | . | . | . | . | . | . | . | 1 | 1 | . | . | . | . | . | . | . | . | . | . |
| *Ranunculus brevifolius* Ten. | . | . | . | . | . | . | . | . | . | . | . | . | . | . | . | . | . | . | 1 | . | 1 | . | . | . | . | . | . | . | . | . | . | . | . |
| *Trinia dalechampii* (Ten.) Janch. | . | . | . | . | . | . | . | . | . | . | . | . | . | . | . | . | . | . | . | . | 1 | . | . | . | . | . | + | . | . | . | . | . | . |
| *Saxifraga sedoides* L. subsp. *sedoides* | . | . | . | . | . | . | . | . | + | . | . | . | . | . | . | . | . | . | . | . | . | . | . | . | 1 | . | . | . | . | . | . | . | . |
| *Alchemilla alpina* L. | . | . | . | . | . | . | . | . | . | . | . | . | . | . | . | . | . | . | . | . | . | . | . | . | . | . | . | . | . | . | + | . | . |
| *Anthyllis montana* L. subsp. *montana* | . | . | . | . | . | . | . | . | . | . | . | . | . | . | . | . | . | . | . | . | . | . | . | . | . | . | . | . | + | . | . | . | . |
| *Arabis alpina* L. subsp. *caucasica* (Willd. ex Schlecht.) Briq. | . | . | . | . | . | . | . | . | . | . | . | . | . | . | . | . | . | . | . | . | . | . | . | . | + | . | . | . | . | . | . | . | . |
| *Aster alpinus* L. | . | . | . | . | . | . | . | . | . | . | . | . | . | . | . | . | . | . | . | . | . | . | . | . | . | . | . | . | . | + | . | . | . |
| *Astragalus depressus* L. subsp. *depressus* | . | . | . | + | . | . | . | . | . | . | . | . | . | . | . | . | . | . | . | . | . | . | . | . | . | . | . | . | . | . | . | . | . |
| *Barbarea bracteosa* Guss. | . | . | . | . | . | . | . | . | . | . | . | . | . | . | . | . | 3 | . | . | . | . | . | . | . | . | . | . | . | . | . | . | . | . |
| *Bellis pusilla* (N. Terrac.) Pignatti | . | . | . | . | . | . | . | . | . | . | . | . | . | . | . | . | . | . | 1 | . | . | . | . | . | . | . | . | . | . | . | . | . | . |
| *Crepis pygmaea* L. subsp. *pygmaea* | . | . | . | . | . | . | . | . | . | . | . | . | . | . | . | . | . | . | . | . | . | . | . | . | . | + | . | . | . | . | . | . | . |
| *Gentiana brachyphylla* Vill. subsp. *favratii* (Rittener) Tutin | . | . | . | . | . | . | . | . | . | . | . | . | . | . | . | . | . | . | . | . | . | . | . | . | . | + | . | . | . | . | . | . | . |
| *Hieracium villosum* L. | . | . | . | . | . | . | . | . | . | . | . | . | . | . | . | . | . | . | . | . | . | . | . | . | . | . | . | . | . | . | . | + | . |
| *Phyteuma orbiculare* L. | . | . | . | . | . | . | . | . | . | . | + | . | . | . | . | . | . | . | . | . | . | . | . | . | . | . | . | . | . | . | . | . | . |
| *Ranunculus breyninus* Crantz | . | . | . | . | . | . | . | . | . | . | . | . | . | . | . | . | . | . | 1 | . | . | . | . | . | . | . | . | . | . | . | . | . | . |
| *Ranunculus seguieri* Vill. | . | . | . | . | . | . | . | . | . | . | . | . | . | . | . | . | . | . | . | . | . | . | . | . | . | . | . | . | . | 1 | . | . | . |
| *Saxifraga italica* D.A. Webb | . | . | . | . | . | . | . | . | . | . | . | . | . | . | . | . | . | . | . | . | . | . | . | . | . | . | . | 1 | . | . | . | . | . |
| *Sedum magellense* Ten. subsp. *magellense* | . | . | . | . | . | . | . | . | . | . | . | . | . | . | . | . | . | . | . | . | + | . | . | . | . | . | . | . | . | . | . | . | . |
| *Sibbaldia procumbens* L. | . | . | . | . | . | . | . | . | + | . | . | . | . | . | . | . | . | . | . | . | . | . | . | . | . | . | . | . | . | . | . | . | . |
| *Thesium parnassi* A. DC. | . | . | . | . | . | . | . | . | . | . | . | . | . | . | . | . | . | . | . | . | . | . | . | . | . | . | . | . | . | . | . | . | + |
| *Veronica aphylla* L. | . | . | . | . | . | . | . | . | . | . | . | . | . | . | . | . | . | . | . | . | . | . | . | + | . | . | . | . | . | . | . | . | . |
| *Veronica fruticans* Jacq. | . | . | . | . | . | . | . | . | . | . | . | . | . | . | . | . | 1 | . | . | . | . | . | . | . | . | . | . | . | . | . | . | . | . |

Rel. 1: Manzini refuge, N 4660389,73, E 424777,76 (2014/07/15); Rel. 2: Manzini refuge, N 4660381,23, E 424811,42 (2014/07/15); Rel. 3: Manzini refuge, N 4660429,87, E 424708,72 (2014/07/15); Rel. 4: Manzini refuge, N 4660254,12, E 424858,62 (2014/07/15); Rel. 5: Femmina Morta Valley, N 4659171,76, E 424531,24 (2014/07/16); Rel. 6: Femmina Morta Valley, N 4658830,23, E 424882,6 (2014/07/16); Rel. 7: Femmina Morta Valle, N 4658283,09, E 424808,21 (2014/07/16); Rel. 8: Femmina Morta Valley, N 4657845,89, E 424907,74 (2014/07/16); Rel. 9: Femmina Morta Valley, N 4657579,23, E 425045,25 (2014/07/16); Rel. 10: Femmina Morta Valley, N 4657400, E 425047,95 (2014/07/16); Rel. 11: Femmina Morta Valley, N 4656072,85, E 425209,85 (2014/07/16); Rel. 12: Fondo di Femmina Morta, N 4655336,47, E 425533,22 (2014/07/16); Rel. 13: Fondo di Femmina Morta, N 4655242,19, E 425426,61 (2014/07/16); Rel. 14: Fondo di Femmina Morta, N 4655114,44, E 425431,31 (2014/07/16); Rel. 15: Fondo di Femmina Morta, N 4654930,91, E 425366,49 (2014/07/16); Rel. 16: Fondo di Femmina Morta, N 4654850,24, E 425388 (2014/07/16); Rel. 17: Fondo di Femmina Morta, N 4655018,83, E 425223 (2014/07/16); Rel. 18: Forchetta Maiella, N 4655074,43, E 424980,33 (2014/07/16); Rel. 25: Tre Portoni, N 4660804, E 424584 (2014/08/05); Rel. 26: Tre Portoni, N 4660632, E 424500 (2014/08/05); Rel. 31: Between Piano Amaro and Femmina Morta Valley, N 4658712, E 425140 (2014/08/05); Rel. 32: Femmina Morta Valley, N 4658694, E 424853 (2014/08/05); Rel. 33: Femmina Morta Valley, N 4659143, E 424637 (2014/08/05), Rel. 27: Mt. Amaro, N 4660208, E 424196 (2014/08/05); Rel. 28: Mt. Amaro, N 4659784, E 424528 (2014/08/05); Rel. 29: Mt. Amaro, N 4659717, E 424556 (2014/08/05); Rel. 30: Mt. Amaro, N 4659646, E 424775 (2014/08/05), Rel. 19: Pesco Falcone, N 4661920,5, E 426728,35 (2014/08/04); Rel. 20: Cima Pomilio, N 4661136,227, E 426111,2 (2014/08/04); Rel. 21: Between Cima Pomilio and Mt. Rotondo, N 4661260, E 425761 (2014/08/04); Rel. 22: Between Cima Pomilio and Mt. Rotondo, N 466129Z, E 425712 (2014/08/04); Rel. 23: Tre Portoni, N 4660563, E 424616 (2014/08/05); Rel. 24: Tre Portoni, N 4660870, E 424701 (2014/08/05).
